# Malignant Perivascular Epithelioid Cell Neoplasm of Left Kidney Treated With Targeted Therapy: A Rare Case Report

**DOI:** 10.7759/cureus.43097

**Published:** 2023-08-07

**Authors:** Muhammad Haseeb, Priyanka Sachdev, Mary Sravani, Chandana Tadigotla, Naga Anjani Bhaskar Srinivas Sunkara, Nikhil Gadyalpatil

**Affiliations:** 1 Internal Medicine, Allama Iqbal Medical College, Lahore, PAK; 2 Internal Medicine, Liaquat University of Medical and Health Sciences, Jamshoro, PAK; 3 Internal Medicine, SVS Medical College, Hyderabad, IND; 4 Internal Medicine, P.E.S Institute of Medical Science and Research, Kuppam, IND; 5 Oncology, SVS Medical College, Hyderabad, IND

**Keywords:** sarcomas, tuberous sclerosis, renal neoplasms, mtor inhibitors, epithelioid cell tumors

## Abstract

Perivascular epithelioid cell neoplasm (PEComa) is one of the rare entities which is challenging to diagnose clinically. These tumors occur due to tuberous sclerosis complex gene mutations leading to upregulation and overexpression of the mammalian target of rapamycin (mTOR). Malignant PEComas are rare, and we report a peculiar case of PEComa treated with mTOR inhibitors. A 43-year-old woman presented with complaints of back pain, intermittent fever, dysuria, and cough with expectoration for one month. Abdominal computed tomography (CT) revealed heterogeneously enhancing exophytic mass of the left kidney. A positron emission tomography CT whole body showed a primary malignancy in the left kidney, sclerotic lesions in the bony skeleton, and lymphangitis carcinomatosis in both lungs. A biopsy of the left renal mass revealed PEComa, focally positive for melanocytic and muscle markers. She was commenced on treatment with intravenous temsirolimus, and there was a complete tumor regression by the end of the completion of six cycles.

## Introduction

Perivascular epithelioid cell neoplasms (PEComas) are mesenchymal tumors with perivascular clear cell and epithelioid features that co-expresses melanocytic and muscle markers [1]. PEComa encompasses a rare subgroup of sarcoma which include groups of tumors like angiomyolipoma (AML), clear-cell “sugar” tumor (CCST) of the lung and extrapulmonary sites, lymphangioleiomyomatosis (LAM), clear-cell myomelanocytic tumor of the falciform ligament/ligamentum teres and rare clear-cell tumors of other anatomical sites [2]. PEComas exhibit a peculiar morphology and are characterized by myomelanocytic tumor markers expression.

PEComas are seen to arise more in middle age and have a marked female predominance of 7:1 [2]. They have been seen to arise most commonly in the gynecologic tract, pelvis, and retroperitoneum and can be of varying malignant potential, but they can occur anywhere in the body. PEComas, particularly AMLs, and LAM, are among the typical physical manifestations of tuberous sclerosis complex (TSC) called Bourneville-Pringle disease [3].

These tumors have been seen to be associated with TSC, an autosomal dominant genetic disease due to losses of the genes *TSC1* (9q34) or *TSC2* (16p13.3), which have also been identified in PEComas [3,4]. These genetic alterations activate the mammalian target of rapamycin (mTOR) in adenosine monophosphate-activated protein kinase (AMPK) and mitogen-activated protein kinase pathways (MAPK), resulting in aberrantly high mTOR activity, which increases TSC-associated AML and PEComas [5-8]. Most PEComas are benign; however, some PEComas show malignant behavior as evidenced by size, lack of circumscription, nuclear atypia, and uncontrolled metastasis [6]. There is no universally effective therapy against PEComas today; however, mTOR inhibitors have shown efficacy in a few studies [8,9]. Hereby, we present a malignant metastatic PEComa of a kidney treated with mTOR inhibitor.

## Case presentation

**Figure 1:**
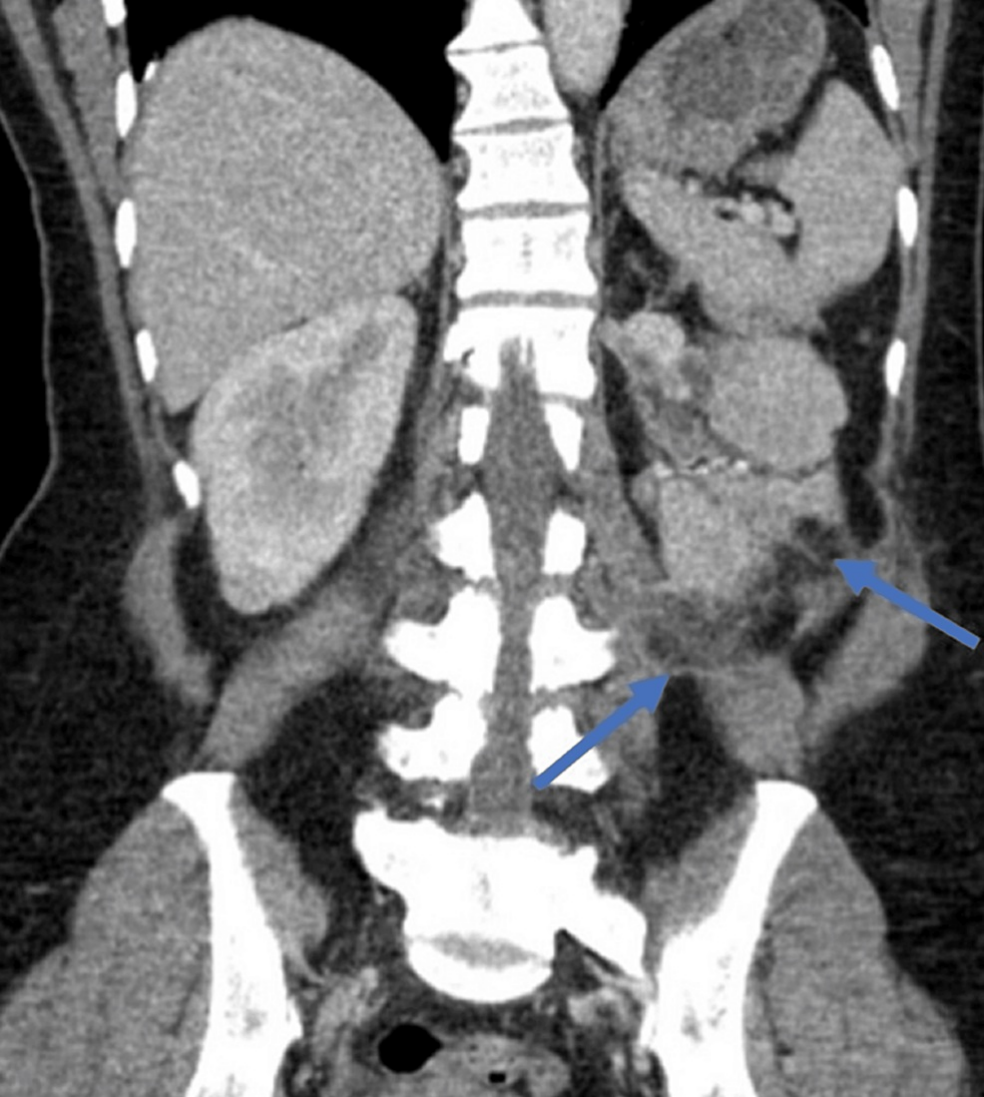
CT abdomen demonstrating an enhancing lesion in the left kidney (blue arrows).

**Figure 2:**
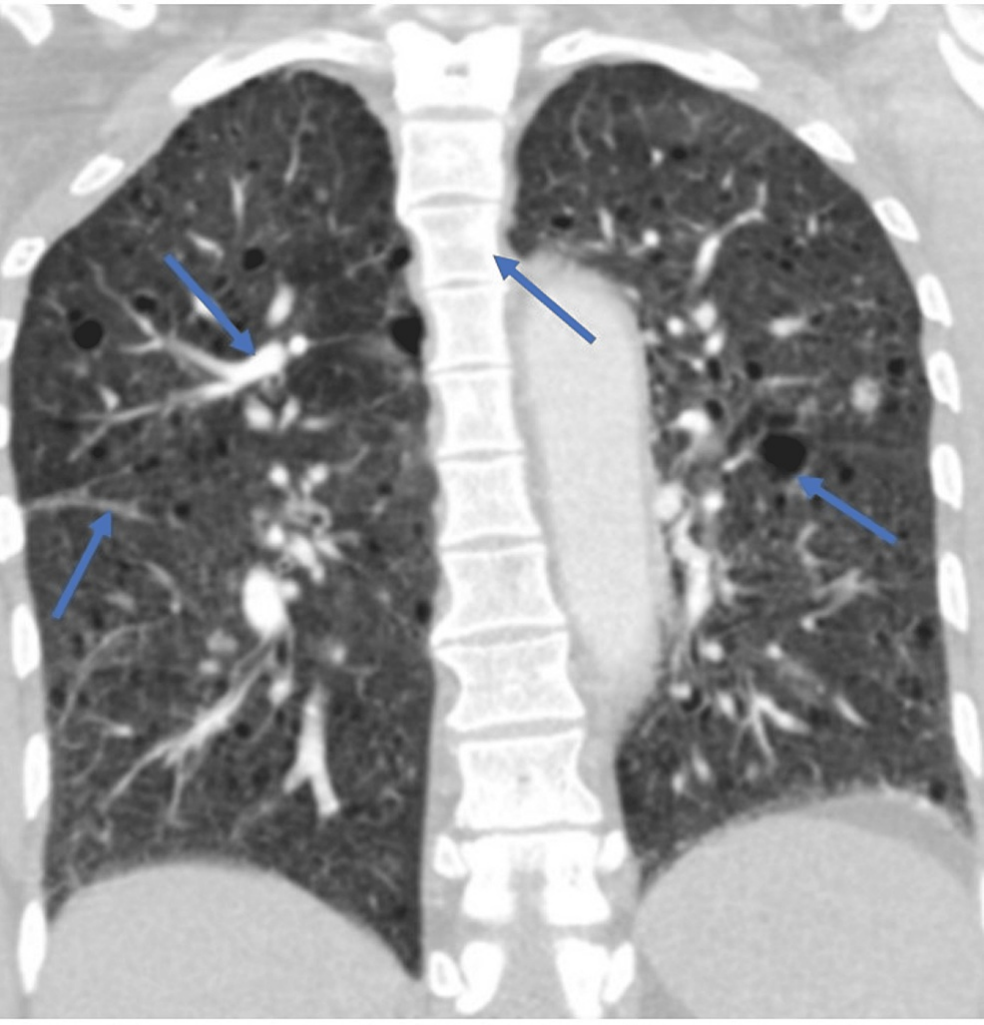
CT chest showing multiple pulmonary nodules, ill-defined opacities, numerous thin-walled parenchymal cysts and sclerotic lesions of the vertebral bodies (blue arrows). CT: computed tomography.

**Figure 3:**
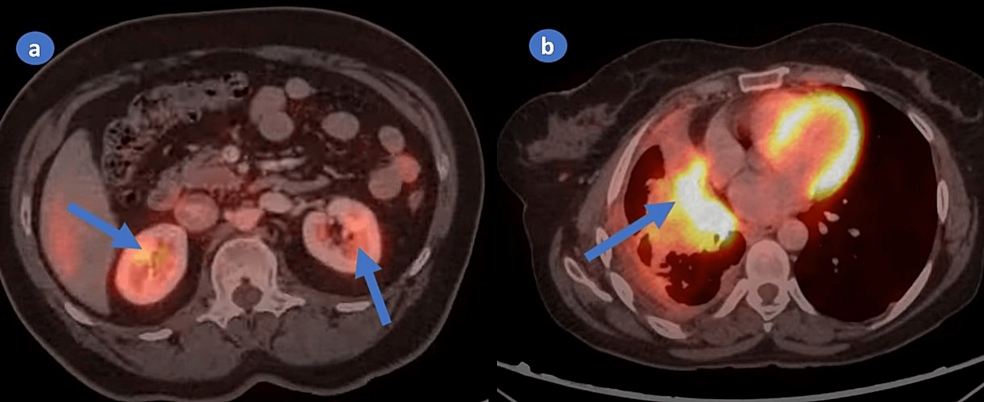
PET-CT revealing enhanced tumor activity in kidneys (a) and lungs (b). PET-CT: positron emission tomography-computed tomography.

**Figure 4:**
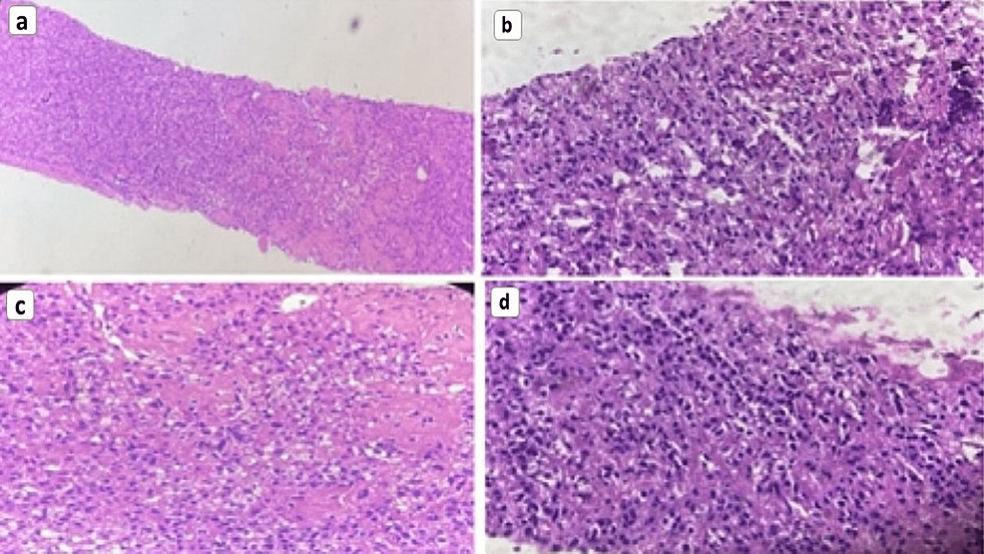
Left kidney biopsy on histopathology demonstrating severe atypia, high mitotic activity, cytoplasmic pleomorphism, bundles of atypical mitotic figures (a-c), and marked coagulative necrosis (d).

A 43-year-old woman presented to the hospital with complaints of back pain, intermittent fever, and dysuria, cough with expectoration for one month. She had no history of smoking, alcohol or substance abuse. A computed tomography (CT) abdomen was performed because of the above symptoms. It showed well-circumscribed oval to round heterogeneously enhancing exophytic mass lesions measuring approximately 76 × 35 × 68mm and 36 × 33 × 27mm with central enhancing areas involving the upper and mid pole of the left kidney, respectively (Figure 1). Multiple sclerotic lesions were noted in the dorso-lumbar spine, sacrum, pelvic bones, bilateral ribs, sternum, and slightly prominent para-aortic, external iliac, and inguinal lymph nodes. CT chest revealed multiple pulmonary nodules, ill-defined opacities, numerous thin-walled parenchymal cysts, and sclerotic lesions of the vertebral bodies (Figure 2). For further evaluation, positron emission tomography whole body (PET-WB) was done, suggesting primary malignancy in the left kidney, sclerotic lesions in the skeleton, and lymphangitic carcinomatosis in both the lungs and right kidney (Figures 3a, 3b). Biopsy from the left renal mass revealed PEComa. On biopsy, the tumor cells were focally positive for a marker of melanocytic differentiation human melanoma black 45 (HMB45), desmin, smooth muscle actin (SMA), and vimentin and negative for pan-CK (cytokeratin), paired box gene 8 (Pax8), and common acute lymphocytic leukemia antigen (CALLA/CD45); occasional lymphovascular emboli were also noted within the tumor (Figures 4a-4d).

Therefore, a diagnosis of malignant PEComa of the kidney with metastasis was made, and the patient was started on treatment with intravenous (IV) temsirolimus on a two-weekly schedule. Post first dose, she had a dramatic improvement in her general condition. She completed six doses of IV temsirolimus which she tolerated well. The patient responded well to the treatment administered and was completely asymptomatic after completing six cycles.

Follow-up PET-WB post-treatment showed a complete metabolic response with interval reduction in the size of lesions in the left kidney and significant reduction in the extent of lymphangitic carcinomatosis in both lungs and stable skeletal lesions, suggestive of an overall good response to the administered treatment. Following this, she was started on an oral mTOR inhibitor, tablet everolimus, 10 mg, and was kept on monthly follow-up. She developed grade-2 stomatitis, occasional headaches, hypercholesterolemia, fluctuating blood sugars, and elevated blood pressure recordings (systolic range: 110-170 mmHg) and was treated symptomatically during her follow-up.

## Discussion

PEComa has been defined as “a mesenchymal tumor composed of histologically and immunohistochemically distinctive perivascular epithelioid cells.” PEComas are considered ubiquitous tumors and have been described in various organs. Criteria for classifying these tumors as “benign”, “of uncertain malignant potential”, and “malignant” have been proposed. A significant association between tumor size >5 cm, infiltrative growth pattern, high nuclear grade, necrosis, and mitotic activity >1/50 high power field with subsequent aggressive clinical behavior of PEComas has been found. PEComas are defined by the immunohistochemical (IHC) co-expression of myoid markers (SMA, desmin, caldesmon) and melanocytic markers (HMB-45, Melan-A, MiTF). Expression varies with morphology: tumors with predominant spindle cell morphology show strong expression of muscle markers and limited expression of melanocytic markers; predominantly epithelioid tumors may strongly express melanocytic markers with limited muscle marker expression. Recently, cathepsin K, a transcriptional target of the MiTF family, emerged as a sensitive marker for PEComa [10].

Malignant PEComas are rare; hence, large-volume studies have not been feasible. Consensus on the treatment of this disease has also been lacking. Prior to the availability of mTOR inhibitors, metastatic PEComas were treated with chemotherapy and vascular endothelial growth factor (VEGFR) inhibitors with varying results [11]. Acknowledgment of the usage of mTOR inhibitors first came to light by Wagner et al. 2010, who first published a report with a series of three patients diagnosed with metastatic cancer [12]. Additionally, a study done by Benson et al. on malignant PEComa from a series of 25 patients with disease types of AML and LAM treated with sirolimus for 12 months has shown promising results [13].

We have summarized the relevant studies on 79 PEComa patients treated with treatment modalities such as chemotherapy, surgery, VEGFR, and mTOR inhibitors (Table 1) [13-16]. The following studies weigh the effectiveness of various treatment options, from chemotherapy or surgery to mTOR inhibitors. So, responses from these studies need to be considered before starting treatment for rare diseases like PEComas. The median age of these patients lies between 26 and 76 years. These studies include 58 females and 21 males with the disease most commonly involving the gynecological tract, kidney, gastrointestinal tract, retroperitoneal sites, bone, trunk, and other Visceral organs. Data include 79 patients, of which 51 were treated with mTOR inhibitors, 27 with chemotherapy, 12 with VEGFR inhibitors as a second- and third-line treatment, and one underwent surgery and had been treated with sirolimus due to tumor recurrence following surgery.

**Table 1:** Relevant studies conducted on PEComa treatment. NR: not reported, m: month, d: day, RECIST: response evaluation criteria in solid tumor, PR: partial response, N/A: not available, SD: stable disease, PD: progressive disease, CR: complete response, PFS: progression-free survival.

Study	No. of patients	Treatment	Treatment duration	Response	Side effects	RECIST1.1 response	Survival rate (%)	PFS (months)	Median follow-up (months)
GenBenson et al. [13]	10	Sirolimus, temsilorimus	7 d-45.5 m	Good (7), No response (3)	Hypertriglyceridemia, hyperglycemia, thrombocytopenia	PR (5), N/A (3), SD (1), PD (1)	78.80%	26 m	21 m
Sanlippo et al. [14]	53	Chemotherapy (23), VEGFR, mTOR inhibitors (40), sirolimus (32), everolimus (5), temsirolimus (3)	24 m	Good	NR	CR (1), PR (15), SD (14), PD (9)	41%	9 m	32.5 m
Gennata et al. [15]	1	Surgery, imatinib, everolimus	10 m	Good	Stomatitis, nasopharyngitis, acne-like skin lesions	CR	NR	>10 m	37 m
Switaj et al. [16]	15	Sirolimus (11), chemo (4)	25 m	Good (15)	Hypertriglyceridemia, hypercholesteremia	PR (8), SD-= (4), CR (3)	83% (sirolimus), 65% (overall)	42.6 m	55.7 m

Consequently, studies showed that patients treated with mTOR inhibitors have a good response, with survival rates ranging from 41% to 83%. The treatment duration differs from seven days to 45.5 months, with progression-free survival ranging between 4.9 months and 42.6 months, as seen in our case. The most common adverse effects seen in these studies are hypertriglyceridemia, hyperglycemia, thrombocytopenia, stomatitis, and skin lesions. So far, studies have provided firm evidence that mTOR inhibitors provide more benefits than other treatment options [13-16]. The role of surgical resection and chemo- and radiotherapy is currently poorly defined. However, locally advanced or metastatic disease portends a poor prognosis, and strategies incorporating chemotherapy, radiation, and immunotherapy have been reported. There are obvious difficulties in performing a therapeutic trial, mainly due to the rarity of the disease. Furthermore, the potential benefits of adjuvant chemotherapy have not been investigated [17]. Limited studies and other single case reports have also shown similar responses. Likewise, we treated our patient initially with IV temsirolimus followed by oral everolimus [15]. She showed an excellent response and is seen to have regressing disease on follow-up and is completely asymptomatic now.

## Conclusions

PEComas are mesenchymal neoplasms with a perivascular appearance on histopathology. This case highlights a potential case of malignant PEComa treated with mTOR inhibitors. Our case also underlines the importance of early diagnosis and management to avoid significant morbidity and mortality. Although the efficacy of mTOR inhibitors has been established in treating malignant PEComa with rare side effects, further studies on a larger scale are warranted with regular follow-up.
